# An Account of Two Remarkable Indian Dwarfs

**Published:** 1851-06

**Authors:** J. Mason Warren


*An account of two remarkable Indian dwarfs, exhibited in Boston, under the name of the Aztec children.* By J. Mason Warren, M. D.

Most of our readers, probably, have seen notices in the newspaper prints of the *Aztec children*, which have been exhibited in the eastern cities. Dr. Warren has drawn up an able, and highly interesting description of their appearance, and anatomical conformation, illustrated by two fine plates, after drawings by Dr. John C. Dalton, junior. The account is contained in the American Journal of Medical Sciences, for April 1851, and has been republished in a pamphlet form.

We quote the following general description:

"The children are a boy and girl, and from the appearance offered by their dentition, hereafter to be given, the former is from seven to eight years of age, the latter from four to six; allowance being made for a retarded condition of these organs, on account of the otherwise abnormal want of development of the whole body. The boy is thirty-three and three-quarters inches in height, and his weight twenty and three-eighths pounds. The girl is twenty-nine and a half inches high, and her weight seventeen pounds. Their skin is of a dark yellowish cast, lighter than what is generally attributed to the Indian in this part of the country, and somewhat darker than that of the mulatto. The hair at the middle parting rises at an inch distant from the root of the nose, but on each side a fine hair descends quite to the edge of the orbit. In the boy, it is black, coarse, and quite stiff — in the girl, wavy and curled. The eyes are large and lustrous. The nose of the boy is quite prominent, and as seen in profile somewhat arched, but seen in front it is a little flattened at the apex; the nostrils are expanded, this feature being less marked in the girl than in the boy. The line of the nostril is oblique, instead of being longitudinal as in the Caucasian race. The separation of the cartilages at the apex is not easily distinguished. The supra-orbitar ridges are very prominent, the head receding directly behind. There are no superciliary prominences or tubercles. In the boy, a ridge, with its convexity towards the meridian line, extends from the external angular process of the frontal bone along the edge of the parietal bone, and nearly joins the edge of the occipital ridge. The occipital bone is much flattened from behind forwards. The continuation of the sagittal suture through the frontal bone to the ossa nasi, corresponding with the fœtal division, is also elevated into a ridge in the male, but not in the female. A circumstance of some interest is the situation of the external auditory foramen, which is much more in a line with the orbit than usual, a fact I have observed in some small heads of low intelligence. There are no indications that artificial compression has ever been used.

In both the children, the upper jaw projects considerably below the lower, the mouth remaining partly open in the boy from a dropping of the lower jaw, which leaves the teeth exposed.

The combination of these two circircumstances, connected with a slight escape of the saliva, which may be partly attributed to the irritation caused by dentition, gives a more unintelligent expression to the face when at rest than it would otherwise have. The upper lip is large, and appears swollen as in strumous subjects. The chin is receding.

The anatomical proportions of the girl seem to be in most respects as perfect as could be desired; with regard to the boy, the following are worthy of notice. The forearm is generally maintained in a slightly bent position, and in a state of semi-pronation, permitting neither entire extension nor perfect supination, forming laterally an external obtuse angle with the arm. The little finger is malformed, being shorter than usual, its tip extending only a little beyond the middle joint of the adjacent one; the last joint is inflexible, and the natural folds on the back of the phalanges, which denote its position are wanting. A slightly webbed appearance is given to the fingers by an increased development of the interphalangeal folds of skin. The hand itself is quite short, broad, and thick.

With regard to the organs of generation, there is a slight malformation of the penis, the urethral aperture being more open than usual, thus approximating to hypospadias. The frenum is wanting. The testicles have not descended into the scrotum, and cannot be distinguished in the groin.

The position generally assumed by these children is peculiar, and may well be compared to that of some of the Simian tribe. The head, particularly in the boy, is thrown forward, as if placed more in advance on the spine than usual. This is accompanied with a slight stoop of the shoulders, and bending of the knees, the whole attitude being well delineated in the graphic sketches by Dr. Dalton. The motion is unsteady, as in the tribe of animals already referred to, the boy having a swinging gait, not unlike that of a person slightly intoxicated."

The measurements of the skeleton, of some parts of the body, are given, which we omit.

The writer adds:

"It may not be uninteresting to state that these children were vaccinated, first the boy, and eight days after the girl was vaccinated from her brother. The disease took well, and went through the usual normal stages. About three weeks after the vaccination, both were attacked on the same day with chicken pox, which pursued a perfectly regular course, and was unattended with any strongly-marked constitutional symptoms.

A question naturally arises to an observer first visiting these beings, whether they belong to the human species, and it is only after the eye becomes accustomed to their appearance that the brotherhood is acknowledged.

I will not here enter into a description of their appearance: it is rather agreeable, in a degree intelligent, and with nothing repulsive, as would be expected in the usual abnormal specimens of the human race. They are both quite apt to comprehend what is said to them, particularly if accompanied by appropriate gestures, although any continued conversation evidently could not be understood. They are, in fact without any language of their own. They seem to acquire words readily, and since their sojourn in Boston, have learned to repeat a number, such as "Papa," "Mamma," "Ellen," "Take care," &c., and evidently are capable of instruction to a limited extent. They are quite imitative, and in this respect nothing escapes them. With regard to any communication by signs or language which they may have with each other, it appears to be at present not much greater than what might be expected from two intelligent individuals of the canine race, although in the expression of their feelings they occasionally make use of an unintelligible jargon of sounds together, which by some might be interpreted as an attempt at language.

As to their habits, they are those of children of two or three years of age, requiring the care of superiors to feed and clothe them. The propensity to constant feeling may also be considered as remarkable, and although at present under the intelligent management of the person who has them in charge, their diet and regimen have been reduced to a system; yet, if left to their own inclinations, they would undoubtedly keep themselves filled with food. With the exception of a catarrhal affection, which might be expected from their exposure to a cold climate, their health seems good; and their strength, as manifested by an almost incessant movement from morning till night, is not to be complained of."

After instituting a comparison with the measurements of some small heads, those belonging to infants, idiotic children, and the heads of the quadrumana, approximating most nearly to man; and presenting sketches of three of the most celebrated dwarfs of whom history furnishes authentic accounts, viz., *Babet Schreier*, *Jeffrey Hudson*, and *Bebe*, the writer concludes that although of low mental organization, they cannot be pronounced idiots of the lowest grade. Their degree of intelligence has decidedly improved since their arrival at Boston, and their capacity for education appears far greater than in the lowest idiots. The fact, universally allowed by physiological writers, that dwarfs are impotent with individuals of ordinary height, and among themselves, is opposed to the idea that these singular creatures belong to any peculiar dwarfish tribe.

The children are exhibited as belonging to a race of dwarfs, the descendants of priests, from a hitherto undiscovered city in Central America. Dr. W. says, that " the peculiar form of their heads, so exactly represented in the travels of Mr. Stevens, as carved on some of the monuments in that region, and those on some of the Egyptian relics, seemed to favor this idea, as it was supported by a most ingenious and romantic story, descriptive of their discovery and transportation to America." He adds, " It is now pretty well understood that they belong to some of the mixed tribes of Indians inhabiting Central America, and we hope hereafter to procure some exact details as to the peculiarities of their parents."

